# High creatinine clearance in critically ill patients with community-acquired acute infectious meningitis

**DOI:** 10.1186/1471-2369-13-124

**Published:** 2012-09-27

**Authors:** Alexandre Lautrette, Thuy-Nga Phan, Lemlih Ouchchane, Ali AitHssain, Vincent Tixier, Anne-Elisabeth Heng, Bertrand Souweine

**Affiliations:** 1 Departments of Intensive Care Unit and Nephrology, University hospital of Clermont-Ferrand, Clermont-Ferrand, France; 2 Univ Clermont 1, UFR Médecine, Clermont-Ferrand, F-63001, France; 3 Departments of Biostatistics, University hospital of Clermont-Ferrand, Clermont-Ferrand, France

**Keywords:** Critically ill, Glomerular filtration rate, High creatinine clearance, Meningitis

## Abstract

**Background:**

A high dose of anti-infective agents is recommended when treating infectious meningitis. High creatinine clearance (CrCl) may affect the pharmacokinetic / pharmacodynamic relationships of anti-infective drugs eliminated by the kidneys. We recorded the incidence of high CrCl in intensive care unit (ICU) patients admitted with meningitis and assessed the diagnostic accuracy of two common methods used to identify high CrCl.

**Methods:**

Observational study performed in consecutive patients admitted with community-acquired acute infectious meningitis (defined by >7 white blood cells/mm^3^ in cerebral spinal fluid) between January 2006 and December 2009 to one medical ICU. During the first 7 days following ICU admission, CrCl was measured from 24-hr urine samples (24-hr-UV/P creatinine) and estimated according to Cockcroft-Gault formula and the simplified Modification of Diet in Renal Disease (MDRD) equation. High CrCl was defined as CrCl >140 ml/min/1.73 m^2^ by 24-hr-UV/P creatinine. Diagnostic accuracy was performed with ROC curves analysis.

**Results:**

Thirty two patients were included. High CrCl was present in 8 patients (25%) on ICU admission and in 15 patients (47%) during the first 7 ICU days for a median duration of 3 (1-4) days. For the Cockcroft-Gault formula, the best threshold to predict high CrCl was 101 ml/min/1.73 m^2^ (sensitivity: 0.96, specificity: 0.75, AUC = 0.90 ± 0.03) with a negative likelihood ratio of 0.06. For the simplified MDRD equation, the best threshold to predict high CrCl was 108 ml/min/1.73 m^2^ (sensitivity: 0.91, specificity: 0.80, AUC = 0.88 ± 0.03) with a negative likelihood ratio of 0.11. There was no difference between the estimated methods in the diagnostic accuracy of identifying high CrCl (p = 0.30).

**Conclusions:**

High CrCl is frequently observed in ICU patients admitted with community-acquired acute infectious meningitis. The estimated methods of CrCl could be used as a screening tool to identify high CrCl.

## Background

The glomerular filtration rate (GFR) can affect the pharmacokinetic/pharmacodynamic relationships of drugs eliminated by the kidney. The dosages and intervals of administration of these drugs are routinely adjusted in patients with a diminished GFR to achieve effective treatment and to limit drug-induced toxicity. GFR is currently estimated in clinical practice by creatinine clearance (CrCl). CrCl is recommended in most textbooks and guidelines to decide on the dosage of drugs with kidney clearance for patients with renal insufficiency. In most studies CrCl is measured from 24-hour urine samples (24-hr-UV/P creatinine) or estimated according to Cockcroft-Gault formula and the simplified Modification of Diet in Renal Disease (MDRD) equation [1].

High CrCl has been reported in groups of intensive care unit (ICU) patients in the post-operative setting [2], and in patients with burns [3] or suffering from cerebral lesions [4]. In patients admitted to the ICU with community-acquired infectious meningitis, prompt and adequate anti-infective treatment is mandatory and high drug dosage administration is advocated [5] to achieve effective anti-infective agent concentrations in the cerebral spinal fluid (CSF). Some of these anti-infective agents undergo kidney clearance. However, the presence of high CrCl in this group of patients is particularly interesting because it may allow the physician to adopt specific anti-infective dosing strategies and thereby avoid treatment failure and drug-induced resistance [6]. The major aim of the study was to record the rate of high CrCl, calculated by 24-hr UV/P creatinine, in patients admitted to the ICU with community-acquired infectious meningitis. The secondary aim was to assess the accuracy of Cockcroft-Gault formula and the simplified MDRD equation in identifying high CrCl.

## Methods

### Study patients

We reviewed the charts of all consecutive patients older than 18 years admitted between January 2006 and December 2009 to the 9-bed medical ICU of the University Hospital of Clermont-Ferrand (France) for community-acquired acute infectious meningitis. A computerized search tool was used to identify patients from the hospital discharge diagnostic database. Meningitis was defined by CSF pleocytosis > 7 white blood cells × 10^6^/L, as proposed by other investigators [7]. Patients with meningitis due to a documented non-infectious cause or that had occurred during hospitalization or within one week after discharge were excluded. Patient characteristics, clinical features, results of investigations, treatment, and outcome were extracted from medical records. The item “diabetes” corresponded to patients receiving a specific medication for diabetes before hospital admission. The Simplified Acute Physiology Score (SAPS) II score and the Sequential Organ Failure Assessment (SOFA) score on the admission-day were calculated from the charts.

### Assessment of creatinine clearance

In our ICU, the patient’s weight, 24-hour urine output, serum and urine creatinine levels are daily measured as standard practice. All serum creatinine samples are measured by the hospital biochemistry laboratory, using a compensated Jaffe method (Modular P, Roche Diagnostics GmbH, Mannheim, Germany). No change in clinical practice was made during the study period. CrCl was determined daily during the first 7 days of ICU stay by one measured method, 24-hr-UV/P creatinine, and two estimated methods, Cockcroft-Gault formula and the simplified MDRD equation (Additional file 1: Table S1) [1]. The CrCl measured by 24-hr UV/P creatinine and estimated by Cockcroft’s equation was normalized by body surface area, as recommended by KDOQI guidelines [1]. The height and weight used for calculation of body surface area were measured on ICU admission. The patients’ height and weight were measured in a supine position with a tape measure and a Hill–Rom bed (Medicraft, Marrickville NSW, Australia), respectively. High CrCl was defined as CrCl >140 ml/min/1.73 m^2^ by 24-hr-UV/P creatinine.

### Statistical analysis

High CrCl was expressed as incidence, in which the numerator is the number of patients with at least one measurement of high CrCl and the denominator the overall number of patients. The percentage of days with a high CrCl during the first 7 days of ICU stay was expressed as duration ratio, in which the numerator is the total number of days with high CrCl and the denominator the total number of ICU days of the overall population. High CrCl incidence and duration ratio were therefore expressed as a percentage. Continuous variables, presented as mean ± SD or as median (IQR), were compared by Mann–Whitney U test. Categorical variables were compared by Fisher’s exact test.

For comparisons between measurements methods, we built a Bland-Altman plot between differences and averages seeking possible biases and outliers. The diagnostic accuracy of Cockcroft-Gault formula and the simplified MDRD equation in predicting high CrCl was then assessed by measuring the area under the receiver operating characteristic (ROC) curves. Each measure was treated as an independent event. The areas under the ROC curves were compared by the Wilcoxon rank test. The best threshold with their corresponding likelihood ratios (negative and positive) was defined by Youden’s index. Statistical tests were performed with the SAS program except for the AUC, which was assessed by Medcalc. A value of p < 0.05 was considered to be significant.

## Results

During the study period, 1266 patients were admitted to the ICU. Of these, 32 had community-acquired acute infectious meningitis. No cases were excluded because of missing data. The characteristics of the patients and the causative organisms of meningitis are shown in Tables 1 and 2, respectively. In 18 patients bacterial or fungal meningitis was diagnosed by a positive CSF culture, and in 2 patients viral meningitis was diagnosed by a positive CSF viral polymerase chain reaction test result. In 12 patients the diagnosis of community-acquired acute infectious meningitis was considered in spite of the absence of a causative organism. Of these, 7 were receiving antibiotics prior to lumbar puncture. In the 12 patients, the mean CSF glucose level, protein level and white cells count was 5.0 ± 2.4 mmol/L, 1.1 ± 1.4 g/L, and 47 ± 53 × 10^6^/L, respectively.

**Table 1:** Characteristics of the patients

**Characteristics**	**N = 32**
Gender ratio (men/women)	15/17
Age, yrs^a,b^	54 ± 16
Weight, kg^a,b^	71 ± 25
Height, cm^a,b^	166 ± 11
Body surface area, m^2^, ^a,b^	1.77 ± 0.27
Diabetes, n (%)^b^	6 (19)
SAPSII, pts^a,b^	46 ± 19
SOFA, pts^a,b^	7 ± 4
Neurologic SOFA, pts^a,b^	2 ± 1
Mechanical ventilation, n (%)^c^	22 (69)
Vasopressive drug, n (%)^c^	13 (41)
Renal replacement therapy, n (%)^c^	3 (9)
ICU length of stay, days^d^	13 (8-19)
ICU mortality, n (%)	4 (12)

^a^: mean ± SD; ^b^: on ICU admission; ^c^: during ICU stay ; ^d^: median (IQR).

**Table 2:** Causative organisms of meningitis

	**N = 32**
Bacteria	
Streptococcus pneumoniae, n	9
Other strepotococci, n	3
Staphylococcus aureus, n	2
Mycobacterium Tuberculosis, n	1
Escherichia coli, n	1
Virus	
Herpes virus type 6, n	1
Cytomegalovirus, n	1
Parasite	
Cryptococcus neoformans, n	2
Indeterminate, n	12

**Figure 1:**
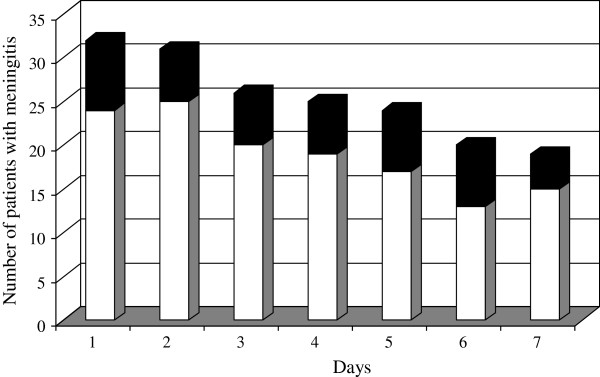
**Evolution of the daily frequency of high CrCl in patients with community-acquired acute infectious meningitis. ***Black box*, number of patients with high CrCl. *White box*, number of patients without high CrCl.

The incidence of high CrCl was 47% (15/32) during the 7-day study period, and the duration ratio was 26%. On ICU admission, the frequency of high CrCl was 25% (8/32). The daily frequency of high CrCl was unchanged throughout the first 7 days of ICU stay, (P = 0.60) (Figure 1). Except for SAPS II on admission, there were no differences in the characteristics of the population upon admission and during ICU stay irrespective of the presence or absence of high CrCl (Additional file 2: Tables S2 and Additional file 3: Table S3).

**Figure 2:**
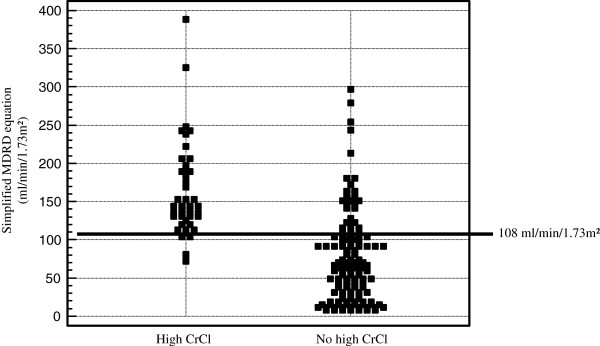
Distribution of creatinine clearance values performed with simplified MDRD equation according of the diagnosis of high CrCl.

For the diagnosis of high CrCl, the area under the ROC curves of Cockcroft-Gault formula was 0.90 ± 0.03 and the simplified MDRD equation was 0.88 ± 0.03. Between Cockcroft-Gault formula and the simplified MDRD equation, there was no difference in the diagnostic accuracy identifying high CrCl (P = 0.30). For Cockcroft-Gault formula, the best threshold was 101 ml/min/1.73 m^2^, corresponding to a sensitivity of 96% and a specificity of 75%. For the simplified MDRD equation, the best threshold was 108 ml/min/1.73 m^2^ with a sensitivity of 91% and a specificity of 80%. Bias, as illustrated by the mean difference in the Bland and Altman analysis between 24-hr-UV/P creatinine and Cockcroft-Gault formula and between 24-hr-UV/P creatinine and the simplified MDRD equation was 0.9 and −3.1 ml/min/1.73 m^2^ respectively. The differences, as illustrated by the ± 95% fluctuation interval in the Bland–Altman graphs, between 24-hr-UV/P creatinine and Cockcroft-Gault formula and between 24-hr-UV/P creatinine and the simplified MDRD equation were −99.7 to 101.4 ml/min/1.73 m^2^ and −113.1 to 106.9 ml/min/1.73 m^2^, respectively (Additional file 4: Figures S1A-B). Four of the 44 measurements with high CrCl had a value of simplified MDRD equation under the threshold of 108 ml/min/1.73 m^2^ from ROC curve analysis (Figure 2). The likelihood ratio of a negative result was 0.11, meaning that the odds of high CrCl is 10 times smaller when the value of simplified MDRD equation is under the threshold of 108 ml/min/1.73 m^2^.

**Figure 3:**
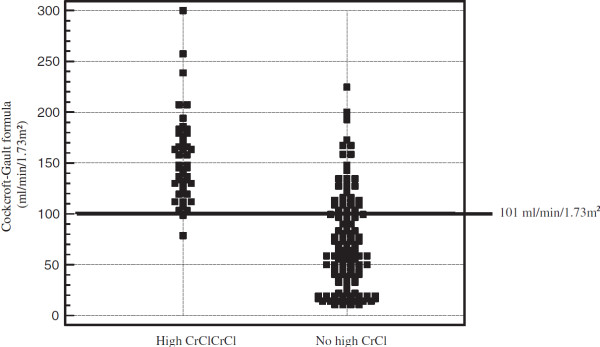
Distribution of creatinine clearance values performed with Cockcroft-Gault formula according of the diagnosis of high CrCl.

Of the 44 measurements with high CrCl, 2 had a value of Cockcroft-Gault formula under the threshold of 101 ml/min/1.73 m^2^ from ROC curve analysis (Figure 3). The likelihood ratio of a negative result was 0.06, meaning that the odds of high CrCl is more than 10 times smaller when the value of Cockcroft-Gault formula is under the threshold of 101 ml/min/1.73 m^2^. There was no difference in the 15% and 30% accuracy between Cockcroft-Gault formula and the simplified MDRD equation for diagnosing high CrCl, 36% vs 20% (P = 0.17) and 68% vs 52%, (P = 0.19), respectively.

## Discussion

Our study showed that high CrCl is often seen on admission in ICU patients with community-acquired acute infectious meningitis. It can persist for several days and be predicted by the estimated methods.

CrCl is classically screened for GFR despite limitations. Creatinine is metabolised and secreted by the renal tubule, and hence CrCl both overestimates the GFR and delays the diagnosis of acute kidney injury. Inulin clearance is the gold standard test for accurately measuring GFR. However it is complex to perform, highly expensive, and cannot therefore be routinely used in ICU patients. Iohexol clearance is an alternative to estimating GFR. However, the use of iodine in the test probably precludes its sequential and repeated use in patients and in healthy volunteers. In clinical practice, despite its limitations, CrCl is currently used to estimate GFR because it is inexpensive, harmless, and easy to perform, particularly in ICU patients. Moreover, it is now well established that small plasma creatinine variations are independently associated with poor prognosis in ICU patients [8], reinforcing the widespread measurement of plasma creatinine for the diagnosis and follow up of kidney function in these patients.

There is no consensus on what constitute normal GFR values, but most authors consider them to range between 90 and 130 ml/min/1.73 m^2^[9]. Similarly, the normal values of CrCl are not agreed upon and most authors extrapolate them from those defined for GFR. Several threshold values have been used to define high CrCl, ranging from 120 ml/min/1.73 m^2^ to 150 ml/min/1.73 m^2^ for women and 160 ml/min/1.73 m^2^ for men [10-13]. In light of all these factors, we chose a 140 ml/min/1.73 m^2^ threshold to define high CrCl. This probably allows a greater specificity in the diagnosis of high CrCl than in most studies. We made no distinction between male and female patients since gender entails no difference in treatment, in particular with regard to dosage. Cockcroft-Gault formula and simplified MDRD equation are commonly used to determine CrCl because diuresis quantification and urinary creatinine measurement are not necessary in the equations. Hence, estimation of CrCl by these two methods is simpler than calculation by 24-hr UV/P creatinine. CrCl as estimated from equations has been recommended for dosage adjustment in patients with reduced kidney function receiving antibiotics with renal elimination. However, no consensus firmly states which methods must be used in ICU patients to determine CrCl. In our study we defined high CrCl as clearance above 140 ml/min/1.73 m^2^ by 24-hr UV/P creatinine. As reported elsewhere, our results demonstrate that CrCl calculated with the 24-hr UV/P creatinine and estimated from equations may yield different results [3,12,14]. Baptista et al. reported that in patients with augmented CrCl, the estimated methods of CrCl significantly under-estimate the measured value of the calculated method [12]. Our findings are in agreement with previously published data and suggest that the estimated methods of CrCl could be used as a screening tool to diagnose high CrCl with a 101 ml/min/1.73 m^2^ threshold for Cockcroft-Gault formula and a 108 ml/min/1.73 m^2^ threshold for simplified MDRD equation. The simplified MDRD equation and the Cockcroft-Gault formula make it possible to rule out a high CrCl when their values are under their respective thresholds.

There is a paucity of information in the literature relating to the characteristics of high CrCl in ICU patients. The incidence of high CrCl in a general population of ICU patients can vary between 18% [11] and 41% [12]. The main factors associated with high CrCl in these studies are young age, greater body surface area, higher diastolic blood pressure, diuresis levels, lower APACHE III scores at admission and multiple trauma [10,11]. In a small sample of patients with head trauma, the incidence of high CrCl was 85% [13]. In addition, some studies that did not specifically deal with high CrCl but either with the assessment of renal function in particular ICU patient groups (burns, trauma, brain injury, surgery) [2-4,10], or with the pharmacokinetic and pharmacodynamic properties of antibiotics [15-18], recorded an incidence of high CrCl ranging from 3% to 50% and values between 130 and 200 ml/min/1,73 m^2^.

Little is known about the pathological mechanisms of high CrCl in ICU patients. The condition has been reported during pregnancy, following nephrectomy, at the onset of diabetic kidney disease, in obesity, in sickle cell anaemia [19] and in children with haemopathy [20]. It has been suggested that high CrCl occurs as a result of glomerular hyperperfusion [21]. Experimental studies have shown that high CrCl is observed during the initial hyperkinetic phase of sepsis [22,23]. However, this hypothesis cannot account for all cases of high CrCl in acute or chronic situations. Another suggested explanation for high CrCl when it is detected at the onset of diabetic kidney disease is that hyperglycaemia stimulates the re-absorption of sodium in the proximal tubule thereby decreasing its supply to the distal tubule. This would set off tubuloglomerular feedback in the macula densa and result in dilatation of the afferent arteriole and an increase in GFR [24]. A third explanation, in particular in children with malignancies and in obese patients is that high CrCl is due to the kidney adjusting to the high protein load caused by large tumours [20] or to the hypermetabolism brought on by obesity [25]. No single hypothesis of the above three seems to explain the high CrCl observed in our study, and it is likely that several mechanisms occur in combination.

In drugs with kidney clearance, a decrease or increase in normal GFR can have a major impact on its plasma concentration. Lowered GFR impairs elimination of the drug and can lead to excessively high concentrations if the dosage is not decreased or if the intervals between doses are not lengthened. Drug overdosage can have serious side effects resulting in increased morbidity [15,26] and for this reason there are dosage guidelines to be followed in the event of a decrease in GFR. Conversely, high GFR can lead to greater concentrations of the drug being eliminated with the possible risk of insufficient dosage and hence treatment failure or, in the case of antibiotics, the emergence of resistant bacteria. There are currently no guidelines for adjusting drug dosage when GFR is high. Our study is not able to assess the pharmacokinetic or pharmacodynamic effects of a drug during high CrCl. Assessments of these effects elsewhere have shown that there is a close connection between the plasma concentration of a drug and CrCl and that, for example, high CrCl is associated with low plasma concentrations of the drug [15,16,27-29]. This suggests that high CrCl could lower drug concentration to a level that renders it therapeutically ineffective, despite a seemingly appropriate dosage. No study has shown a causal relation between high CrCl and failure of drug treatment. Our study shows that high CrCl can persist for several days. It is possible that when high CrCl lasts only a few hours it has little impact clinically whereas a longer period of high CrCl would probably require daily dosage adjustment. It would be interesting to perform a daily monitoring of the presence and intensity of high CrCl in patients taking drugs excreted by the kidneys.

Our study has several limitations. First, it was retrospective and therefore we cannot rule out the possibility that certain eligible patients were excluded because of an oversight in the computer rating of diagnosis of meningitis. However, the main outcome criterion, the incidence of high CrCl, did not influence the selection of the patients recruited. Second, it was a monocentric study including only ICU patients admitted for meningitis, and so extrapolating the results to a general ICU population remains speculative. The study was focused on patients with meningitis, since in this clinical situation, prompt antiinfectious treatment with both adequate agent and dosage is mandatory for preventing poor outcome. High CrCl may impact the pharmacokinetics of the drugs resulting in therapeutic failure due to low drug concentration despite an apparent appropriate drug dosage. Third, the diagnosis of meningitis was based on CSF white cell count, and therefore may have been overestimated. However, this diagnostic criterion is classically used in studies on meningitis in ICU patients and the magnitude of the results would not have been different if we had limited the study to patients with meningitis defined by a CSF culture yielding a microorganism. Fourth, CrCl rate can be affected by certain drugs, such as cimetidine [30] and trimethoprim [31], that stimulate tubular secretion of creatinine. No such drugs were administered in our cohort.

## Conclusions

Half of ICU patients with community-acquired acute infectious meningitis have high CrCl, which, in many cases, may persist for several days. High CrCl can be ruled out when a value of the Cockcroft-Gault formula is under a threshold of 101 ml/min/1.73 m^2^ or when a value of the simplified MDRD equation formula is under a threshold of 108 ml/min/1.73 m^2^. There is a need to assess the clinical impact of high CrCl in ICU patients.

## Abbreviations

GFR: Glomerular filtration rate; CrCl: Creatinine clearance; MDRD: Modification of diet in renal disease; ICU: Intensive care unit; CSF: Cerebral spinal fluid; SAPS: Simplified Acute Physiology Score; SOFA: Sequential Organ Failure Assessment; ROC: Receiver operating characteristic; AUC: Area under the ROC curve; KDOQI: Kidney disease outcomes quality initiative; APACHE: Acute physiology and chronic health evaluation.

## Competing interests

The authors declare that they have no competing interests.

## Authors’ contributions

TNP collected data. LO performed the statistical analysis. AAH, VT and AEH reviewed the intellectual content. AL and BS conceived of the study, drafted the manuscript and performed the statistical analysis. All authors read and approved the final manuscript.

## Pre-publication history

The pre-publication history for this paper can be accessed here:

http://www.biomedcentral.com/1471-2369/13/124/prepub

## Supplementary Material

Additional file 1 **Table S1.** Formula used to determine creatinine clearance and body surface area. Click here for file

Additional file 2 **Table S2.** Demographic data of patients suffering from meningitis with or without high CrCl. Click here for file

Additional file 3 **Table S3.** Causative organisms of meningitis in patients with or without high CrCl. Click here for file

Additional file 4 **Figure S1.** Bland and Altman analysis of 24-hr-UV/P creatinine and the Cockcroft-Gault formula (A) and of 24-hr-UV/P creatinine and the simplified MDRD equation (B). Click here for file
