# Supplementary material for: High creatinine clearance in critically ill patients with community-acquired acute infectious meningitis
Source: BMC Nephrol. 2012 Sep 27;13:124. doi: 10.1186/1471-2369-13-124 (PMC3502432; doi:10.1186/1471-2369-13-124)
Supplement: Additional file 1 — Table S1. Formula used to determine creatinine clearance and body surface area. [file 1471-2369-13-124-S1.doc]

**Additional file**

**Table 1. Formula used to determine creatinine clearance and** body surface area

| 24-hr UV/P creatinine | CrCl = [(UO × Ucr)] / [Scr × 1440] |
| --- | --- |
| Cockcroft-Gault formula | CrCl = [(140 − age) × weight] × k / [Scr]  k=1.2 in males and 1.04 in females |
| Simplified MDRD equation | CrCl (ml/min/1.73m²) = 175x (0.742 if female) x (1.212 if black) x Scr^-1.153^ x age^-0.203^ |
| BSA | BSA (m²) = 0.007184 x Weight^0.425^ x Height^0.725^ |

BSA, body surface area; CrCl, creatinine clearance; 24-hr UV/P creatinine, creatinine clearance measured from 24-hour urine sample; MDRD, modification of diet in renal disease; Scr, serum creatinine concentration (micromole/L), Ucr, urine creatinine concentration (micromole/L); UO, 24 hour urine output (mL)
