# Supplementary material for: High creatinine clearance in critically ill patients with community-acquired acute infectious meningitis
Source: BMC Nephrol. 2012 Sep 27;13:124. doi: 10.1186/1471-2369-13-124 (PMC3502432; doi:10.1186/1471-2369-13-124)
Supplement: Additional file 2 — Table S2. Demographic data of patients suffering from meningitis with or without high CrCl. [file 1471-2369-13-124-S2.doc]

**Table 2. Demographic data of patients suffering from meningitis with or without high CrCl**

| Characteristics | Patients | |
| --- | --- | --- |
|  | No high CrCl (n=17) | High CrCl  (n=15) |
| Gender ratio (men/women) | 8/9 | 7/8 |
| Age, yrs^a^ | 58 ± 15 | 49 ± 15 |
| Weight, kg^a^ | 70 ± 25 | 72 ± 23 |
| Height, cm^a^ | 165 ± 11 | 167 ± 11 |
| Body surface area, m² | 1.75 ± 0.27 | 1.79 ± 0.27 |
| Diabetes, n (%) | 3 (18) | 3 (20) |
| SAPSII admission, pts^a^ | 50 ± 18 | 40 ± 17 |
| SOFA admission, pts^a^ | 7 ± 4 | 6 ± 4 |
| Neurologic SOFA admission, pts^a^ | 2 ± 1 | 3 ± 1 |
| Mechanical ventilation, n (%) | 10 (59) | 12 (80) |
| Vasopressive drug, n (%) | 5 (29) | 8 (53) |
| Renal replacement therapy, n (%) | 3 (18) | 0 (0) |
| ICU length, days^b^ | 13 [8-15] | 15 [8-25] |
| ICU mortality, n (%) | 1 (6) | 3 (20) |

^a^: mean ± SD; ^b^: median [IQR]
