# Supplementary material for: High creatinine clearance in critically ill patients with community-acquired acute infectious meningitis
Source: BMC Nephrol. 2012 Sep 27;13:124. doi: 10.1186/1471-2369-13-124 (PMC3502432; doi:10.1186/1471-2369-13-124)
Supplement: Additional file 3 — Table S3. Causative organisms of meningitis in patients with or without high CrCl. [file 1471-2369-13-124-S3.doc]

**Table 3. Causative organisms of meningitis in patients with or without high CrCl**

|  | Patients | |
| --- | --- | --- |
|  | No high CrCl (n=17) | High CrCl  (n=15) |
| Bacteria |  |  |
| Streptococcus pneumoniae, n | 4 | 5* |
| Other strepotococci, n | 0 | 3* |
| Staphylococcus aureus, n | 2* | 0 |
| Mycobacterium Tuberculosis, n | 1 | 0 |
| Escherichia coli, n | 1 | 0 |
| Virus |  |  |
| Herpes virus type 6, n | 1 | 0 |
| Cytomegalovirus, n | 1 | 0 |
| Parasite |  |  |
| Cryptococcus neoformans, n | 2 | 0 |
| Indeterminate, n | 5 | 7* |

*: one patient is died in the group
