# Supplementary material for: High creatinine clearance in critically ill patients with community-acquired acute infectious meningitis
Source: BMC Nephrol. 2012 Sep 27;13:124. doi: 10.1186/1471-2369-13-124 (PMC3502432; doi:10.1186/1471-2369-13-124)
Supplement: Additional file 4 — Figure S1. Bland and Altman analysis of 24-hr-UV/P creatinine and the Cockcroft-Gault formula (A) and of 24-hr-UV/P creatinine and the simplified MDRD equation (B). [file 1471-2369-13-124-S4.doc]

**Additional file**

**Figure Legends**

**Figure 1.** Bland and Altman analysis of 24-hr-UV/P creatinine and the Cockcroft-Gault formula (**A**) and of 24-hr-UV/P creatinine and the simplified MDRD equation (**B**).

(A)

(B)
